# Social Studies of Science and denialism: implications for public health

**DOI:** 10.1590/0102-311XEN055826

**Published:** 2026-10-30

**Authors:** Kenneth Rochel de Camargo

**Affiliations:** 1 Instituto de Medicina Social Hesio Cordeiro, Universidade do Estado do Rio de Janeiro, Rio de Janeiro, Brasil.

**Keywords:** Science, Disinformation, Public Health, Ciência, Desinformação, Saúde Pública, Ciencia, Desinformación, Salud Pública

## Abstract

This article examines scientific denialism through the lens of the Social Studies of Science. We argue that, while constructionist approaches have been essential for critically analyzing scientific knowledge production, they are insufficient to address the deliberate distortion of science driven by political, economic, and ideological interests. After outlining the historical development of Social Studies of Science and its emphasis on epistemological agnosticism, the paper notes its limitations in confronting intentional campaigns designed to manufacture doubt rather than advance scientific understanding. The article reviews key denialist tactics and provides case studies - tobacco, vaccines, HIV/AIDS, and COVID-19 - that demonstrate how denialism has produced severe public health consequences. The conclusion argues for a balanced position that preserves the critical scrutiny of science while clearly distinguishing good-faith scientific critique from strategically motivated attacks on scientific consensus.

## Resumo

Este estudo examina o negacionismo científico sob a ótica dos Estudos Sociais da Ciência. Argumentamos que, embora as abordagens construtivistas tenham sido essenciais para criticar a produção de conhecimento científico, elas são incapazes de enfrentar a distorção deliberada da ciência motivada por interesses políticos, econômicos e ideológicos. Após delinear o desenvolvimento histórico dos Estudos Sociais da Ciência e sua ênfase no agnosticismo epistemológico, este estudo observa o seu confronto limitado de campanhas que buscam intencionalmente fabricar dúvidas em vez de avançar o entendimento científico. Este estudo revisa táticas negacionistas chave e apresenta estudos de caso - tabaco, vacinas, HIV/aids e COVID-19 - que demonstram como o negacionismo produziu graves consequências para a Saúde Pública. Sua conclusão defende uma posição equilibrada que preserve o escrutínio crítico da ciência e distinga claramente a crítica científica bem-intencionada de ataques estrategicamente motivados ao consenso científico.

## Resumen

Este artículo analiza el negacionismo científico desde la perspectiva de los Estudios Sociales de la Ciencia. Sostenemos que, si bien los enfoques construccionistas han sido esenciales para analizar críticamente la producción de conocimiento científico, resultan insuficientes para abordar la distorsión deliberada de la ciencia impulsada por intereses políticos, económicos e ideológicos. Tras esbozar la evolución histórica de los Estudios Sociales de la Ciencia y su énfasis en el agnosticismo epistemológico, el artículo señala sus limitaciones a la hora de hacer frente a campañas intencionales diseñadas para generar dudas en lugar de promover el conocimiento científico. El artículo analiza las principales tácticas negacionistas y presenta casos de estudio -el tabaco, las vacunas, el VIH/SIDA y la COVID-19- que demuestran cómo el negacionismo ha tenido graves consecuencias para la salud pública. La conclusión aboga por una postura equilibrada que mantenga el escrutinio crítico de la ciencia, al tiempo que distinga claramente entre la crítica científica de buena fe y los ataques con motivaciones estratégicas contra el consenso científico.

## Some definitions

The Social Studies of Science constitute an interdisciplinary field that developed in the mid-1970s, at least in part as a response to a classical sociology of science approach that did not question the core of scientific production, but rather its social circumstances. Robert Merton and his classic work on the values of the scientific community ^1^ are the paradigmatic example of that perspective. He assumed science as an expression of what Ziman called the “received view” ^2^; that is, a naive realist perspective that viewed scientific activity as the discovery of preexisting objects and relationships, a mirror of Nature, according to Rorty ^3^.

This approach, based on empirical studies using historical, sociological, and anthropological methods in dialogue with different philosophical perspectives, in many ways follows a path opened by Thomas Kuhn, according to Callon & Latour ^4^. It questions the simplistic image of a science that produced “truths” and a passive representation of “reality”, bringing human activity in the production of knowledge in this framework to the center of the field of vision.

Despite a wide thematic, theoretical, and methodological dispersion, studies produced in this field share some common characteristics, especially a theoretical perspective that could be called *constructionism*; that is, one that emphasizes the social construction of science, its objects, and theories ^5^. Additionally, it challenges the epistemic monopoly of scientists over the production of valid knowledge ^6^, especially with regard to the interface between science and society in the production of evidence-based public policies.

In turn, these characteristics show a clearly iconoclastic approach, which often adopts, even out of necessity, epistemological agnosticism as its basis. However, albeit an important starting point for a critical approach to science, this approach can affect the analysis of concrete interventions, since decision-making on what to do (or not) as to public health, whether considering individuals or communities, are not equivalent in terms of results, regardless of the adopted course of action.

First, while the Social Studies of Science show that participation in the discussion of such interventions cannot be limited to scientists alone, they do not clearly characterize who else would be qualified to have a voice in these debates, a challenge that Collins & Evans ^6^ have called the problem of extension. Additionally, while iconoclastic, its approach assumes somehow that scientific agents are oriented toward the production of knowledge, even if biased, limited by prejudices, and based on misconceptions held by the very scientists. However, the methods and theories developed by the Social Studies of Science are not adequate to address the explicit and deliberate distortion of science for political, economic, or cultural interests.

According to Proctor ^7^, overcoming this limitation of constructionist approaches to science requires studying the construction not only of knowledge, but also of ignorance, which he called *agnotology*: a focus on “*...ignorance - or doubt or uncertainty - as something that is made, maintained, and manipulated by means of certain arts and sciences*” ^7^ (p. 8).

In fact, several authors have focused on the deliberate production of uncertainty or obfuscation of scientific knowledge, especially with regard to issues related to the environment or public health ^8^ ^,^ ^9^ ^,^ ^10^. However, a warning had already been issued at the very heart of the Social Studies of Science by one of its most prominent authors: Latour ^11^ wondered whether criticism had “lost steam”, lamenting that, in an unexpected reversal, critical tools developed to protect society from ideological claims presented as scientific were being used by obscure interests precisely to undermine confidence in perfectly reasonable and solid scientific production, taking as one of his examples the effort of certain sectors of the economy to sabotage public acceptance of the academic consensus on anthropogenic climate change. McGarity & Wagner’s ^8^ detailed exposition notes two characteristics of these attacks on science. First, even recognizing the difficulty of establishing precise limits for what we call science, philosophers and sociologists in the field would agree that research conducted with a predetermined result would not be science. Second, these authors document how various challenges to established science are not aimed at enabling convergence toward scientific consensus, but rather at prolonging the perception of dissent within and outside the scientific community.

This latter strategy was characterized as denialism by Diethelm & McKee ^12^, who developed a description of the strategies employed to undermine scientific consensus. Based on a review of the related literature, Godulla et al. ^13^ expanded this five-item description into a list describing a total of 11 topics on the subject:

(1) Conspiracy theories (almost every denialist argument invokes one or more conspiracy theories);

(2) Selectivity (cherry-picking, i.e., considering only publications that are consistent with personal biases);

(3) False experts (which can refer to individuals with no specific expertise or with expertise in a different field that is irrelevant to the discussion in question);

(4) Impossible expectations (or moving goalposts, setting new expectations for what scientists should achieve in their research and posing unattainable demands, such as 100% certainty);

(5) Logical fallacies and misrepresentations (such as false analogies, distorting an argument to make it easier to refute, among others);

(6) Manufactured doubt (as expressed in the general definition; some authors have coined the term manufactroversy, combining the words “manufactured” and “controversy” to characterize this strategy);

(7) Misinterpretation, for example, of collected data;

(8) Single study fallacy (emphasizing individual studies that contradict established consensus - see item 2);

(9) Questioning the personal motives of scientists;

(10) Framing issues as threats to individual freedom and presenting mainstream science as emanating from certain philosophical or religious beliefs;

(11) Clinging to historical evidence that has long been disproved.

Examples of these strategies will be presented below, in the description of some specific cases of denialism that have impacted public health.

Based on their review, these authors propose the following definition of denialism: it “*...is the motivated, systematic use of rhetorical tactics with the goal of creating the impression of legitimate debate where there is consensus based on reasoned facts and theories*” ^13^ (p. 144).

The reference to motivated use indicates a central and potentially problematic characteristic for identifying possible cases of denialism, which is intentionality in action. It is not a matter of error or mistake, but of deliberate production of uncertainty for specific purposes, somehow threatened by scientific consensus. This refers to the difficulty in establishing the intentions underlying the actions of individuals or groups ^14^; however, it does not represent an impossibility, but rather the need for sufficiently detailed descriptions that allow for such assessment ^15^ ^,^ ^16^. In specific cases, the possibility of analyzing internal documents, previously inaccessible to the public, has enabled the unequivocal characterization of such intentionality; based on these cases, it is possible to identify characteristics that would constitute deliberate distortion of science, such as economic interests contradicted by certain scientific findings and the shift of the debate from the scientific field itself to the media in general ^17^.

Some cases of denialism, such as in the tobacco industry, exhibit obvious underlying economic interest - withal, that does not mean that any conflict of interest would automatically disqualify research results. However, in other situations, such as the anti-vaccine movement, these interests may not be so obvious and/or may be aligned with other types of motivation, such as ideological ones. In the first case, for example, the promotion of conspiracy theories in the health field has synergistic connections with the so-called alternative therapies ^18^. In the second case, conspiracy theories underlying health denialism have been aligned with far-right libertarianism, which preaches minimal government, the primacy of individual autonomy, and consequently maximum individual freedom, fueled by an ecosystem of think tanks and conservative, free-market institutes engaged in the denial of any science that, if considered seriously, implies the limitation of absolute individual freedom by regulations or policies ^19^.

## The role of social media

Denialism, and even scientific denialism, obviously predates internet-based communication platforms (social networks) and even the internet itself. However, their advent has brought some additional characteristics to interpersonal communications with important repercussions for the spread of denialist content.

First, virtually instantaneous communication over any distance has enabled bringing groups of individuals together on a global scale, giving critical mass and social and political visibility to what were previously isolated voices without much influence, no matter how absurd the ideas they share may be - consider, for example, the whole movement around the idea of a “flat earth”. Second, the economic logic of such platforms is based on constant user engagement, which has come to be known as the attention economy ^20^. On the one hand, the algorithms designed to generate engagement make the content offered to users increasingly uniform, in a positive feedback loop that creates a context that is impervious to divergent views ^21^ ^,^ ^22^. On the other hand, they tend to prioritize messages with the potential to trigger emotional responses, which ultimately leads to the spread of provocative or openly hateful discourse ^23^.

The formation of groups with uniform views characterizes that which Nguyen ^22^ calls epistemic bubbles; that is, a socio-epistemic structure in which other relevant voices have been left out, possibly by accident. These would differ from echo chambers, in which such voices are actively excluded and discredited. Epistemic bubbles could be broken by introducing content that challenges the consensus on which they were structured; however, echo chambers, due to their intentional production, include an additional element, which is the discrediting of information sources that are external to the group, similarly to the modus operandi of cults. This makes it much more difficult for members of such chambers to escape them.

The problem is compounded by intrinsic characteristics of the very human thought, such as the tendency toward confirmation bias, the persistence of first impressions that makes them more difficult to dislodge ^24^, and the so-called Dunning-Kruger effect ^25^ ^,^ ^26^, which leads us to overestimate our own epistemic competence, making it even more difficult to confront denialist content once it has been inculcated in individuals and groups.

Considered in conjunction, the above described characteristics create an extremely favorable environment for the spread and persistence of denialist ideas ^27^ ^,^ ^28^ ^,^ ^29^. Given their inherent intentionality, it is therefore essential to explore the interests underlying their creation and dissemination in order to confront them.

The following sections present concrete examples of denialist strategies with negative repercussions for public health. This choice of examples omits another major contemporary denialist issue: climate change denialism. Despite its clearly relevant implications for public health, the scientific hard kernel of the discussion lies within disciplines outside the specific domain of health, such as climatology, and will thus be omitted due to the author’s lack of specific expertise in this area.

## Tobacco

An industry whose product is clearly harmful to health has obvious economic interests in creating doubts about the robust production of knowledge about such harm. Wendell Potter, a former communications executive at a large health insurance company, exposed the tactics used by this industry to manipulate public opinion, claiming that it created the model for such strategies. According to this author, the tobacco industry “*...is really two industries: One makes and sells cigarettes, and the other engages in a full-time effort to foster doubt about criticisms of tobacco products, encourage distrust of government, manipulate legislative processes, distract people from the negative effects, neutralize and harass opponents, coerce and coordinate allies, undermine scientific and common knowledge - and persuade people that smoking is normal, even helpful, human behavior*” ^30^ (p. 213). This denialist model characterizes, according to this author and to Oreskes & Conway ^10^, a playbook that was later adopted by several other actors.

The first articles demonstrating solid evidence of the health risks of tobacco use were published in 1950 ^31^. Almost immediatelly thereafter, the tobacco industry began to develop strategies seeking to discrediting these findings, aiming to raise all kinds of doubts about them ^14^ ^,^ ^15^ ^,^ ^32^ ^,^ ^33^ ^,^ ^34^. These strategies included funding pseudo-studies designed to contradict epidemiological findings, hiring renowned scientists (who were not experts in the specific areas of these studies) to produce criticism in the media, and even harassing researchers studying the issue ^35^.

However, the overwhelming scientific evidence on the health risks of smoking made these strategies increasingly untenable, leading the industry to change tactics in the late 1970s. Instead of directly combating scientific findings, it began to promote the idea of individual freedom for smokers, adopting a technique called third person ^36^; that is, it presented social actors that were supposedly independent in relation to companies to act as spokespersons for their interests. This included hiring social scientists and philosophers to produce favorable arguments and creating organizations supposedly arising from civil society to defend them ^33^.

However, the accumulated evidence on the risks of passive smoking or secondhand smoke put even this strategy in check. Companies in the sector returned to their previous tactic of discrediting research and researchers, publishing paid articles in an attempt to resist the proposed measures to restrict smoking in confined spaces. However, scientific research on smoking-related health issues was so compelling that anti-smoking legislation progressed in several countries. Thus, the final blow to this strategy was a successful lawsuit in the United States that exposed the inner workings of the industry’s denialist manipulation, culminating in an agreement that forced tobacco product manufacturers to post unequivocal statements about the harms of smoking on their websites ^17^.

However, their defeat in this battle did not represent the end of the war. The industry began to direct its production to economically and politically weaker countries, leading to an increased proportion of smokers in their populations, while reducing it in countries that have established effective regulations, including Brazil. In addition, the emergence of electronic cigarettes/vaping has prompted a new marketing offensive by the tobacco industry, which, ever creative, presents these new products as smoking cessation aids.

## Anti-vaccine

The World Health Organization (WHO) has included vaccine hesitancy or refusal in a list of the top ten threats to global health ^37^. Opposition to vaccines is as old as their very existence, but the contemporary anti-vaccine movement had an important trigger in the unfortunate publication by *The Lancet* of a methodologically flawed, possibly fraudulent study, later removed by the journal after virtually all coauthors requested their removal from the authorship, and which led to the main author (Andrew Wakefield) having his medical license revoked in the United Kingdom ^15^ ^,^ ^38^.

The alleged association between the MMR (measles, mumps, and rubella) vaccine and autism in children, baselessly proposed in that article, gave rise to a worldwide movement that has already led to the reintroduction of infectious diseases in countries where they had been eradicated ^13^ ^,^ ^39^ ^,^ ^40^ ^,^ ^41^. Another harmful effect of this false association was reinforcing an ableist view of autism, contributing to the stigma and discrimination against people with autism.

The COVID-19 pandemic and the consequent introduction of vaccines to combat the emerging disease added new layers of conspiracy theories to those that had already been circulating intensely on the internet for more than three decades ^42^. The anti-vaccine movement mobilizes individualistic and conspiratorial arguments that see governments, scientists, and science itself as the main antagonists, supposedly motivated by interests that are harmful to the public.

A relatively small group of content producers are primarily responsible for spreading and disseminating misinformation about vaccines on the internet. A study by the Center for Countering Digital Hate (CCDH) ^43^ showed that just 12 individuals, including notorious names in health denialism, such as Joseph Mercola and Robert F. Kennedy Jr., were responsible for about two-thirds of the misinformation circulating between February and March 2021.

Misinformation about vaccines repeats a relatively small set of themes, notably including the following ^42^:

Dangerous ingredients: components that are safe for use and present in small quantities in vaccines, or even absent from their composition, such as fetal cells, are presented as terrible threats to health ^44^;

“Vaccine damage”: vaccine side effects are presented as much more frequent than established, or including fanciful problems unrelated to vaccines, often resulting from post hoc ergo propter hoc fallacies present in personal accounts;

Arguments from authority based on questionable material produced by supposed experts: a group of anti-vaccine activists (such as the aforementioned Wakefield or the 12 on the CCDH list) has consistently produced materials based on low-quality research and/or misinterpretations of existing data that fuel public mistrust of vaccines;

The incidence of vaccine-preventable diseases has declined for reasons other than vaccines and/or has not actually declined: misinterpretation of epidemiological data is one of the bases for the false argument that better hygiene has led to the control of these diseases in industrialized countries. In the second case, conspiracy theories about polio, for example, claim that what has actually happened is that the name of the disease has been changed;

There are too many vaccines, given in too short a period of time: an argument unfortunately supported by pediatricians who propose alternative schedules without any study to justify them, leading to a delay in completing the vaccination schedule and a consequent unnecessary prolongation of the risks of infection. Ironically, although the number of vaccines has increased over time, refinements in the production process have actually reduced the total amount of antigens administered ^45^;

“Natural immunity” is better: underestimating the risk of exposure to vaccine-preventable diseases and relying on the fallacy of appealing to nature, some people organize “disease parties” to expose healthy children to sick children, for example, with chickenpox;

Parents (especially mothers) really know best: appeals to intuition that supposedly trumps any evidence to the contrary.

Although the spread of vaccine denialism cannot be attributed exclusively to economic interests, these certainly play an important role. For example, the 12 denialists on the CCDH list profit from their activism, either through the financing of their campaigns or simply through the sale of products and services as alternatives to vaccines or to supposedly “detoxify” vaccinated people. In addition to these interests, there is the individualistic ideological perspective propagated by the contemporary far right.

## HIV/AIDS

Shortly after the initial identification of the new disease, all sorts of conspiracy theories were mobilized, particularly about its cause (the disease was said to be the result of a biological warfare agent created in a laboratory, or there was no agent and its cause was simply “sexual promiscuity”) and treatment (antiretroviral drugs would have no effect or would actually be harmful to health) ^18^ ^,^ ^46^ ^,^ ^47^.

AIDS denialism created a particularly tragic example of repercussions on public health when it was elevated to public policy under the presidency of Thabo Mbeki in South Africa ^7^ ^,^ ^12^ ^,^ ^18^ ^,^ ^48^. Supported, on the one hand, by scientists who, despite their academic credentials, lacked the specific expertise necessary to substantiate their criticism, and allied with proponents of ineffective “natural therapies” ^18^ ^,^ ^49^, Mbeki delayed the adoption of antiretroviral therapy in his country as long as possible, leading to an estimated 300,000 unnecessary deaths ^50^.

As in the case of the anti-vaccine movement, economic motivation is not the only factor, and invoking it may even be counterintuitive, given that the pharmaceutical industry had a clear interest in the consumption of the new class of drugs. However, once again, proponents of alternative treatments played an important role in the process, although other factors, such as Mbeki’s supposedly anti-colonial agenda, may have been even more relevant.

## COVID-19

Perhaps the most visible example of scientific denialism in the health field is the explosion of misinformation that followed the outbreak of the COVID-19 pandemic ^18^ ^,^ ^19^ ^,^ ^28^ ^,^ ^51^ ^,^ ^52^ ^,^ ^53^ ^,^ ^54^ ^,^ ^55^. According to the Director-General of the WHO, Dr. Tedros Adhanom Ghebreyesus: “*We’re not just battling the virus; we’re also battling the trolls and conspiracy theorists that push misinformation and undermine the outbreak response*” ^56^.

In this case, denialism was multifaceted, applying to the disease itself, supposed treatments, and later to the vaccines that were quickly developed. Regarding the disease, some narratives sought to minimize its possible impact (“*It’s just an ordinary flu*”) and deny the need for and/or effectiveness of distancing measures. While other narratives were based on conspiracy theories about the causative virus, which was said to be a biological weapon whose development was attributed to various agents according to the ideology of those who had allegedly produced and propagated them - such as the U.S. government, or the Chinese government, or even Bill Gates ^51^.

Supposed treatments, based on bad science or simply on the will of their proponents, proliferated, especially those based on drugs already available on the market, such as chloroquine and its derivatives or ivermectin, in some cases combined in a “COVID kit” that included multiple substances ^57^ ^,^ ^58^ ^,^ ^59^ ^,^ ^60^. With regard to vaccines, there was a reinforcement of existing denialism, coupled with new layers of conspiracy theories, which proclaimed that vaccines were actually a means of reducing the world population or that they would implant electronic chips into people, which in turn would be activated by the 5G cell phone network, adding to another conspiracy theory and consequent movement against the new technology already in place ^18^ ^,^ ^51^.

The adoption of social distancing measures was opposed by several political actors, combining an interest in avoiding any damage to economic activity with the far-right ideology of maximum individual freedom (although even some thinkers further to the left of the political spectrum have bought into this deception). An important instrument in this fight was a document prepared by the American Institute for Economic Research, a libertarian think tank, called the “Great Barrington Declaration”. This manifesto advocated the free circulation of the virus among the population to create herd immunity - a distortion of an expression used in public health for the collective effects of vaccine immunization - while protecting the most vulnerable without actually specifying how that would be done ^19^.

This proposal was adopted by the Federal Government in Brazil, which attempted to sabotage any social distancing initiative, encountering opposition from some state and municipal governments and the judiciary. The federal policy, which was eugenicist in nature by insinuating that only frail people would be victims of the disease ^52^ ^,^ ^54^ ^,^ ^60^, was based on the intention to preserve economic activity at any cost. To justify such laissez-faire, it was necessary to fuel the illusion of a non-existent therapy, which was supported by an association of doctors politically aligned with the government at the time ^60^, similarly to events in the United States ^18^.

Hostility to vaccines stemmed both from political aspects (the first vaccine to be adopted on Brazilian soil came from a state institution, under the government of a former ally of the president who had become an opponent, in collaboration with Chinese institutions) and from libertarian principles of individual primacy.

Various conspiracy theories fueled unprecedented hostility toward health professionals and managers, especially in Brazil and the United States, even leading to death threats against them and their families. In Brazil, for example, far-right opinion makers created a fantastical version of events in which doctors were allegedly receiving financial incentives to report COVID-19 as the cause of death in unrelated cases. A false news story that was widely circulated recounted the case of a supposed cousin who had died as a result of a truck tire explosion and whose death certificate listed COVID-19 as the cause of death. Because of fictions like this, professionals found themselves threatened by family members of deceased victims so they would not include that cause on death certificates. The United States saw a particularly relevant far-right campaign against Anthony Fauci (which continues to date), a respected scientist and public servant who had already been prominent in the fight against AIDS, due to his public positions in defense of scientific guidelines for combating the pandemic, from social distancing measures to vaccines.

In this case, the consequences of denialism were painfully obvious, especially in Brazil, whose response to the pandemic was rated as one of the worst in the world ^61^, resulting in a total of more than 700,000 deaths from COVID-19, about 13% of deaths from the disease worldwide in a country with less than 3% of the global population ^62^.

## Conclusion

The above examples show situations in which the contribution of science to health measures was contrary to certain interests, motivating the development of denialist strategies to delay, minimize, or even prevent its use.

However, this does not mean in any way that science is infallible or unassailable - quite the contrary. Criticism of science is part of science itself, and, in particular, the critical perspective that the Social Studies of Science provide is fundamental for exposing situations in which scientific arguments are used incorrectly, or even when they are themselves erroneous. This is particularly relevant when health issues are concerned, since interference in people’s lives, both individually and collectively, is inevitable. History provides several examples of how misguided science has proven harmful to health, such as in the development of lobotomy (which even led to a Nobel Prize), or in the initial resistance to including women and children in the diagnosis of what would later be called AIDS, on the assumption that the disease was restricted to men who have sex with men. This criticism, which is necessary and correct, must also consider challenges to scientific thinking that are not intended to improve it or protect people, but are in fact motivated by interests contrary to these values.

Harry Collins & Trevor Pinch, applying the Social Studies of Science logic to medicine in their book *Dr. Golem: How to Think about Medicine*, after examining several cases of the complex relationship between science and medicine (including resistance to vaccination), conclude that: “*Science may be wrong* (...) *but this does not make the opposite view right. In the absence of careful research of the opposite view, science is probably the way to bet. This is even more likely to be the case if science is continually put under scrutiny*” ^63^ (p. 202).

However, it should be noted that a strategy to counter denialism in the public space cannot be limited to “better explaining” scientific issues to the general population. Although providing scientifically grounded assertions in the public debate is a necessary condition, it is far from being sufficient. The naive logic behind this assumption - dubbed the “deficit model” ^64^ ^,^ ^65^ - disregards the core aspect of the problem, which is the lack of trust in the very people and institutions that are supposedly speaking in the name of science ^66^. The erosion of trust in science and scientists in the specific examples above are not some spontaneous sociological phenomenon, but, revisiting Proctor’s agnotology, the final product of organized efforts of interested parties. Thus, in order to counter such efforts, broader political action has to be undertaken, and experts that can mediate between the scientific and general communities - such as those who Collins & Evans ^67^ named interactional experts - certainly have a role in presenting not just the conclusions of scientific research, but also its method and process. However, beyond that, political engagement with relevant communities is essential to rebuild trust, and the history of the engagement of civil society with HIV/AIDS research provides a paradigmatic example of how trust can be (re)built from the ground up ^68^.

## Data Availability

Not applicable.
